# Plasma Proteomic Associations with Alzheimer's Disease Endophenotypes

**DOI:** 10.1002/alz70856_103553

**Published:** 2025-12-26

**Authors:** Eric B. Dammer, Shiva Afshar, Shijia Bian, David A. A. Bennett, Richard Mohs, Douglas W. Beauregard, John Dwyer, Nicholas T Seyfried, Blaine Russell Roberts, Cecelia Manzanares, James J. Lah, Allan I. Levey, Erik C.B. Johnson

**Affiliations:** ^1^ Emory University School of Medicine, Atlanta, GA, USA; ^2^ Goizueta Alzheimer's Disease Research Center, Emory University, Atlanta, GA, USA; ^3^ Rollins School of Public Health, Emory University, Atlanta, GA, USA; ^4^ Rush Alzheimer's Disease Center, Chicago, IL, USA; ^5^ Global Alzheimer's Platform Foundation, Washington, DC, USA

## Abstract

**Background:**

Clinical Alzheimer's disease (AD) is currently characterized by cerebral β‐amyloidosis associated with cognitive impairment. However, most cases of AD are associated with multiple neuropathologies at autopsy. The peripheral protein changes associated with these AD endophenotypes are poorly understood.

**Method:**

We analyzed the plasma proteomes of individuals from four cohorts (*n* = 2,103 participants) to identify proteins and pathways associated with cerebral β‐amyloidosis and cognitive function, as well as multiple other neuropathologies commonly associated with AD.

**Result:**

Plasma proteins positively associated with cerebral β‐amyloidosis were enriched in synaptic and extracellular matrix (ECM) pathways, whereas proteins negatively associated with amyloidosis were enriched in metabolism and proteostasis pathways. Many proteins strongly associated with cerebral β‐amyloidosis were influenced by *APOE* ε4 genotype. Proteins positively associated with cognitive function were enriched in protein translation, vesicular transport, and mitochondrial pathways whereas proteins negatively associated with cognitive function were enriched in inflammation, fatty acid metabolism, and ECM pathways. Analyses in a cohort with paired brain data showed that known neuropathologies could account for only half of proteins associated with cognitive function, and that many plasma proteins associated with these neuropathologies are not strongly correlated to levels in brain.

**Conclusion:**

Multiple biological pathways and processes associated with cerebral β‐amyloidosis, cognitive function, and other AD endophenotypes can be observed in plasma, some of which are likely influenced by peripheral factors. Therapeutic approaches targeting these pathways in the central nervous system or the periphery may modify AD risk or disease progression.

